# Coronavirus-Specific Antibody Cross Reactivity in Rhesus Macaques following SARS-CoV-2 Vaccination and Infection

**DOI:** 10.1128/JVI.00117-21

**Published:** 2021-05-10

**Authors:** Catherine Jacob-Dolan, Jared Feldman, Katherine McMahan, Jingyou Yu, Roland Zahn, Frank Wegmann, Hanneke Schuitemaker, Aaron G. Schmidt, Dan H. Barouch

**Affiliations:** a Center for Virology and Vaccine Research, Beth Israel Deaconess Medical Center, Boston, Massachusetts, USA; b Ragon Institute of MGH, MIT and Harvard, Cambridge, Massachusetts, USA; c Harvard Medical School, Boston, Massachusetts, USA; d Janssen Vaccines and Prevention BV, Leiden, The Netherlands; e Department of Microbiology, Harvard Medical School, Boston, Massachusetts, USA; University of Texas Southwestern Medical Center

**Keywords:** SARS-CoV-2, coronavirus, neutralizing antibodies

## Abstract

Vaccines are being rapidly developed with the goal of ending the severe acute respiratory syndrome coronavirus 2 (SARS-CoV-2) pandemic. However, the extent to which SARS-CoV-2 vaccination induces serum responses that cross-react with other coronaviruses remains poorly studied. Here, we define serum profiles in rhesus macaques after vaccination with DNA- or Ad26-based vaccines expressing SARS-CoV-2 spike protein followed by SARS-CoV-2 challenge, or SARS-CoV-2 infection alone. Analysis of serum responses showed robust reactivity to the SARS-CoV-2 full-length spike protein and receptor binding domain (RBD), both included in the vaccine. However, serum cross-reactivity to the closely related sarbecovirus SARS-CoV-1 spike and RBD was reduced. Reactivity was also measured to the distantly related common cold alphacoronavirus (229E and NL63) and betacoronavirus (OC43 and HKU1) spike proteins. Using SARS-CoV-2 and SARS-CoV-1 lentivirus-based pseudoviruses, we show that neutralizing antibody responses were predominantly SARS-CoV-2 specific. These data define patterns of cross-reactive binding and neutralizing serum responses induced by SARS-CoV-2 infection and vaccination in rhesus macaques. Our observations have important implications for understanding polyclonal responses to the SARS-CoV-2 spike protein, which will facilitate future CoV vaccine assessment and development.

**IMPORTANCE** The rapid development and deployment of SARS-CoV-2 vaccines has been unprecedented. In this study, we explore the cross-reactivity of SARS-CoV-2-specific antibody responses to other coronaviruses. By analyzing responses from nonhuman primates (NHPs) both before and after immunization with DNA or Ad26-vectored vaccines, we find patterns of cross-reactivity that mirror those induced by SARS-CoV-2 infection. These data highlight the similarities between infection and vaccine-induced humoral immunity for SARS-CoV-2 and cross-reactivity of these responses to other CoVs.

## Abstract (precis)

The rapid development and deployment of SARS-CoV-2 vaccines has been unprecedented. In this study, we explore the cross-reactivity of SARS-CoV-2-specific antibody responses to other coronaviruses.

## INTRODUCTION

Severe acute respiratory syndrome coronavirus 2 (SARS-CoV-2), the etiological agent of COVID-19, emerged into the human population in mid-December 2019 in Wuhan, capital of the Hubei province in China, and has since spread globally (1, 2). The rapid expansion of the COVID-19 pandemic has made SARS-CoV-2 therapeutics and vaccine development a global health priority. Two mRNA-based (3, 4) and two adenoviral vector-based (5, 6) vaccines, all targeting the SARS-CoV-2 spike (S) protein have been studied in phase III trials in the United States. Additional vaccines, including protein subunit and inactivated virus-based vaccines, are in phase III trials globally (7–13). Currently, two mRNA-based vaccines and Ad26 have received Emergency Use Authorizations from the United States Food and Drug Administration, and ChAdOx1 has been approved for emergency use in the United Kingdom and European Union (14–16).

There are seven coronaviruses that infect humans: four common cold-causing viruses (hCoVs), which include both alpha- and betacoronaviruses, and three highly pathogenic betacoronaviruses (17). SARS-CoV-2 is the third betacoronavirus with pandemic potential, after SARS-CoV-1 and MERS-CoV, to emerge into the human population within the last two decades (17, 18). The hCoVs include two betacoronaviruses, OC43 and HKU1, which are closely related to SARS-CoV-2, and two alphacoronaviruses, 229E and NL63, which are more distantly related (17). These hCoVs circulate annually and seropositivity for all four hCoVs among adults is estimated to be >70% in the general population; most seroconversion events occur during childhood (19, 20).

The S protein, which mediates host cell attachment and subsequent viral membrane fusion, varies greatly on the amino acid level between common cold-causing hCoVs and their pathogenic relatives (SARS-CoV-1, SARS-CoV-2, and MERS-CoV). The greatest sequence variation is within the receptor binding domain (RBD), while the membrane proximal S2 domain, involved in S trimerization and membrane fusion, is conserved (21). This antigenic diversity between hCoV and SARS-CoV-2 RBDs allows the latter to be used as a highly specific antigen for testing seroconversion (22). In contrast, the full-length S protein is less coronavirus strain specific, with high backgrounds in enzyme-linked immunosorbent assay (ELISA)-based analyses, most likely due to cross-reactive responses to the non-RBD domains from prior exposure to hCoVs (21).

Cross-reactive responses have been extensively studied for other viral infections. For influenza and HIV vaccine design efforts, for example, a goal is to elicit cross-reactive and broadly neutralizing responses capable of protecting against infection (23, 24). Neutralizing antibody responses may prevent initial infection of the target cells, and other immune responses may facilitate immunological control of infection and also prevent disease (24, 25). For SARS-CoV-2, cross-reactive responses may play a key role in protection against SARS-CoV-2 and other future coronaviruses (26, 27). We therefore asked whether SARS-CoV-2 infection or vaccination in rhesus macaques led to cross-reactive antibody responses against SARS-CoV-1 and other hCoVs. We profiled serum from rhesus macaques infected with SARS-CoV-2 or vaccinated with DNA- or Ad26-based vaccines expressing SARS-CoV-2 S protein and then challenged with SARS-CoV-2. We describe detectable cross-reactive binding serum responses to SARS-CoV-1 and, to a lesser extent, other hCoVs.

## RESULTS

### Phylogenetic relationships and amino acid conservation of human-infecting coronaviruses.

**FIG 1:**
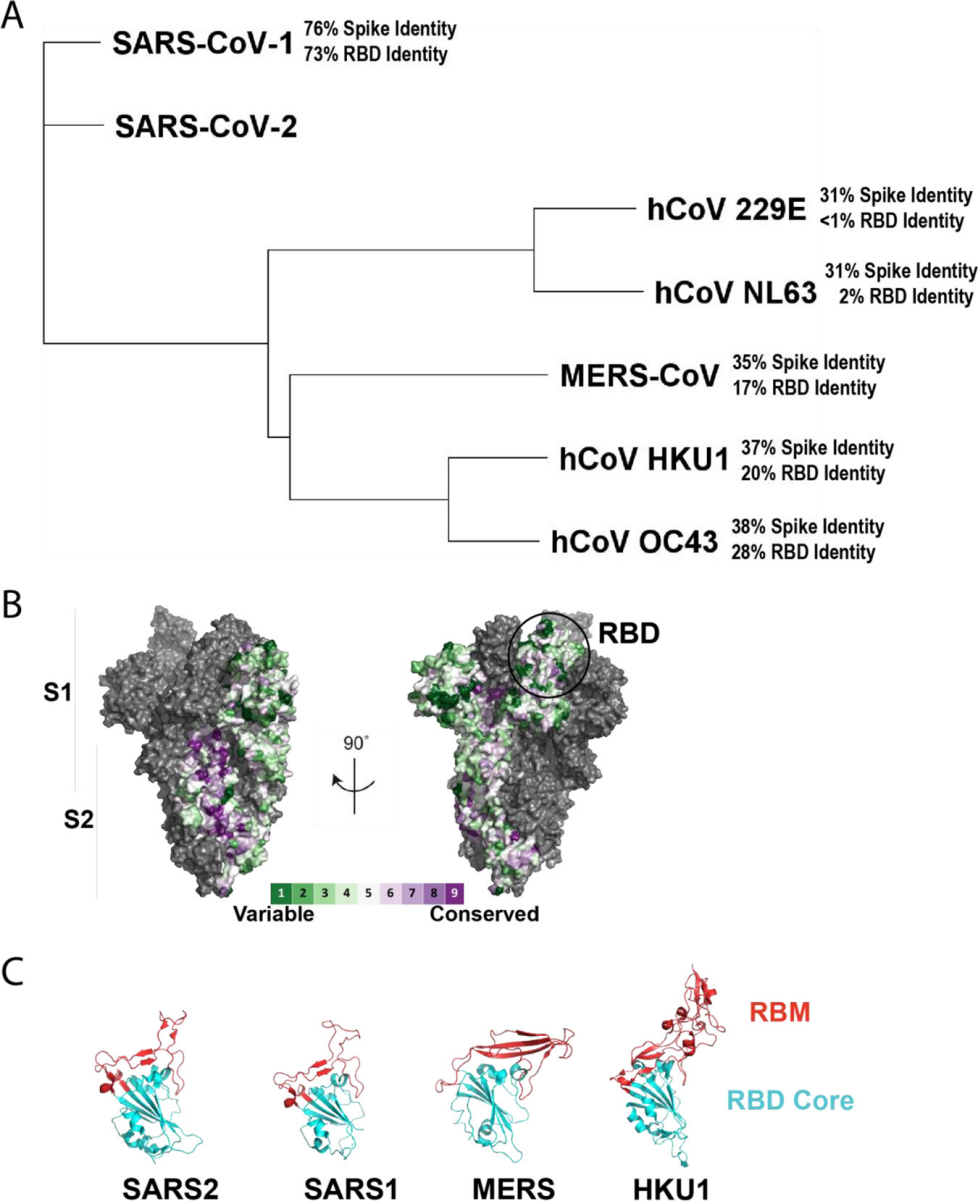
S protein amino acid sequences of human coronaviruses. Representative S protein sequences of each of the seven human infecting coronaviruses were aligned (A) using MUSCLE alignment, and a neighbor-joining tree was generated based on the alignment. The percentage amino acid identity of each of the spike proteins and RBDs is listed in reference to SARS-CoV-2 spike or RBD, respectively. The conservation was plotted as a surface (B) on the structure of SARS-2 S (6VYB) as calculated by the ConSurf database. The receptor binding motifs (RBMs) within the RBD of SARS-CoV-2, SARS-CoV-1, MERS-CoV, and HKU1 are highlighted in (C).

A phylogenetic tree was generated based on representative amino acid sequences of each of the human coronavirus S proteins (Fig. 1A). This sequence alignment was used to generate a conservation map based on the SARS-CoV-2 S structure (Fig. 1B) (28, 29). SARS-CoV-1 is the most closely related to SARS-CoV-2 (76% amino acid identity to SARS-CoV-2 spike), followed by roughly 30 to 38% spike identity to MERS-CoV, HKU1, and OC43, and 31% spike identity to 229E and NL63 (Fig. 1A). Because SARS-CoV-1 and SARS-CoV-2 mainly engage the same receptor, angiotensin-converting enzyme 2 (ACE2), the relatively high degree of spike conservation (76%) and RBD (73%) homology are to be expected (Fig. 1A). While NL63 also engages ACE2, it does so through structurally divergent interactions of its RBD with no apparent structural homology to SARS-CoV-2 or SARS-CoV-1 RBD (21). This is reflected in the phylogenetic tree by minimal (2%) RBD homology of NL63 to SARS-CoV-2 (Fig. 1A). Other CoVs use different entry receptors or attachment factors to facilitate entry into target cells. MERS-CoV, for example, engages DPP4 (dipeptidyl peptidase-4), while HKU1 uses sialic acid as an attachment factor. These different entry or attachment factors likely account for lower RBD (>1 to 28%) conservation compared to full-length S protein (31 to 38%) for each of these viruses compared to SARS-CoV-2 (Fig. 1A and B). Within the RBD, the residues which directly interact with the entry or attachment factor comprise the receptor-binding motif (RBM). The SARS-CoV-2 RBM is structurally similar to the SARS-CoV-1 RBM, and they both engage ACE2. However, the RBM for MERS-CoV is structurally distinct and the RBM for HKU1 contains a large insertion that alters the overall conformation of the RBM relative to the RBD core (Fig. 1C) (30, 31). Because the RBM facilitates engagement of various CoV-specific host cell factors necessary for entry, this likely accounts for the necessary structural differences in the RBMs. The structural similarities, however, raise the question of whether there are cross-reactive antibodies raised by SARS-CoV-2 spike exposure.

### Receptor-binding domain cross-reactivity.

**FIG 2:**
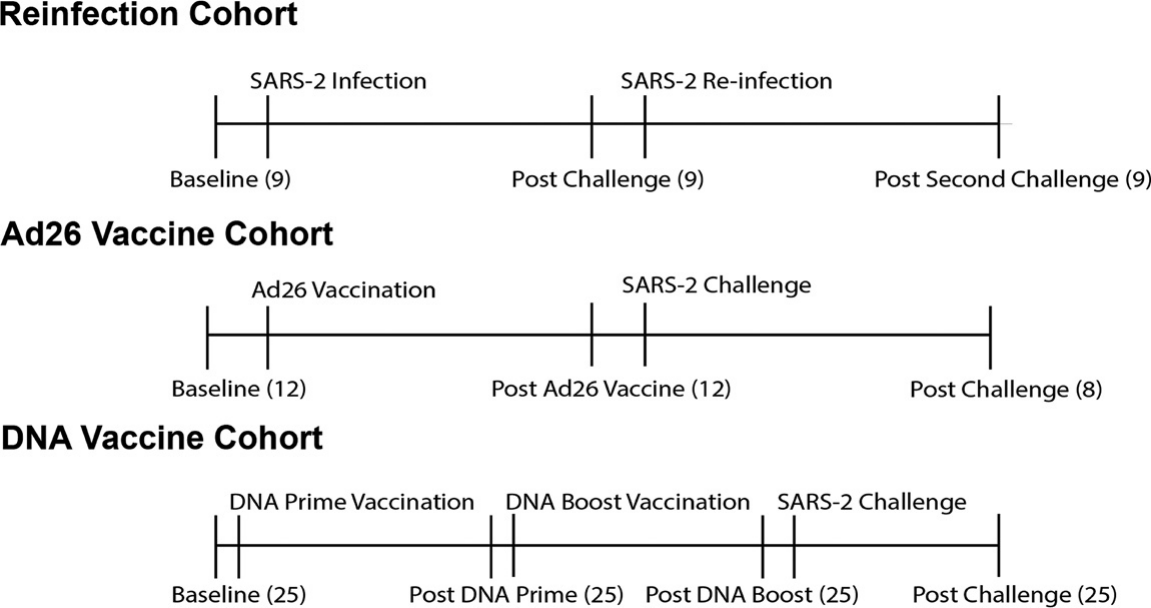
Study schemas. In the reinfection cohort, 9 NHPs were assayed at baseline, infected with SARS-2, assayed, reinfected, and assayed again. In the Ad26 cohort, 12 NHPs were assayed at baseline, vaccinated, and assayed after vaccination, and 8 of these 12 animals were challenged and assayed following challenge. In the DNA vaccine cohort, 25 NHPs were assayed at baseline, primed, assayed, boosted, assayed, challenged, and assayed following challenge. The *n* for each time point is indicated in in parentheses.

Serum and B cell cross-reactivity to the full-length S proteins of highly pathogenic CoVs (e.g., SARS-CoV-1 and MERS-CoV) and circulating common cold hCoVs (e.g., HKU1, OC43, NL63, and 229E) have previously been described in SARS-CoV-2-infected patients (21, 27, 32, 33). Here, we assessed the specificity of cross-reactive responses in SARS-CoV-2-infected and vaccinated rhesus macaques. This study consisted of three nonhuman primate (NHP) cohorts. (i) The first cohort was a reinfection study, with 9 NHPs that were infected with SARS-CoV-2 and allowed to recover and then challenged a second time with SARS-CoV-2 (34) (Fig. 2). (ii) The second cohort contained 12 NHPs vaccinated with Ad26 vaccines expressing various SARS-CoV-2 spike isoforms and 8 of which were challenged with SARS-CoV-2 (35) (Fig. 2). (iii) The third cohort had 25 NHPs vaccinated with DNA vaccines expressing SARS-CoV-2 spike isoforms and boosted with the same DNA vaccine, and then challenged with SARS-CoV-2 (36) (Fig. 2). Sham NHPs were included in each of these cohorts, as previously published (data not shown) (34–36).

**FIG 3:**
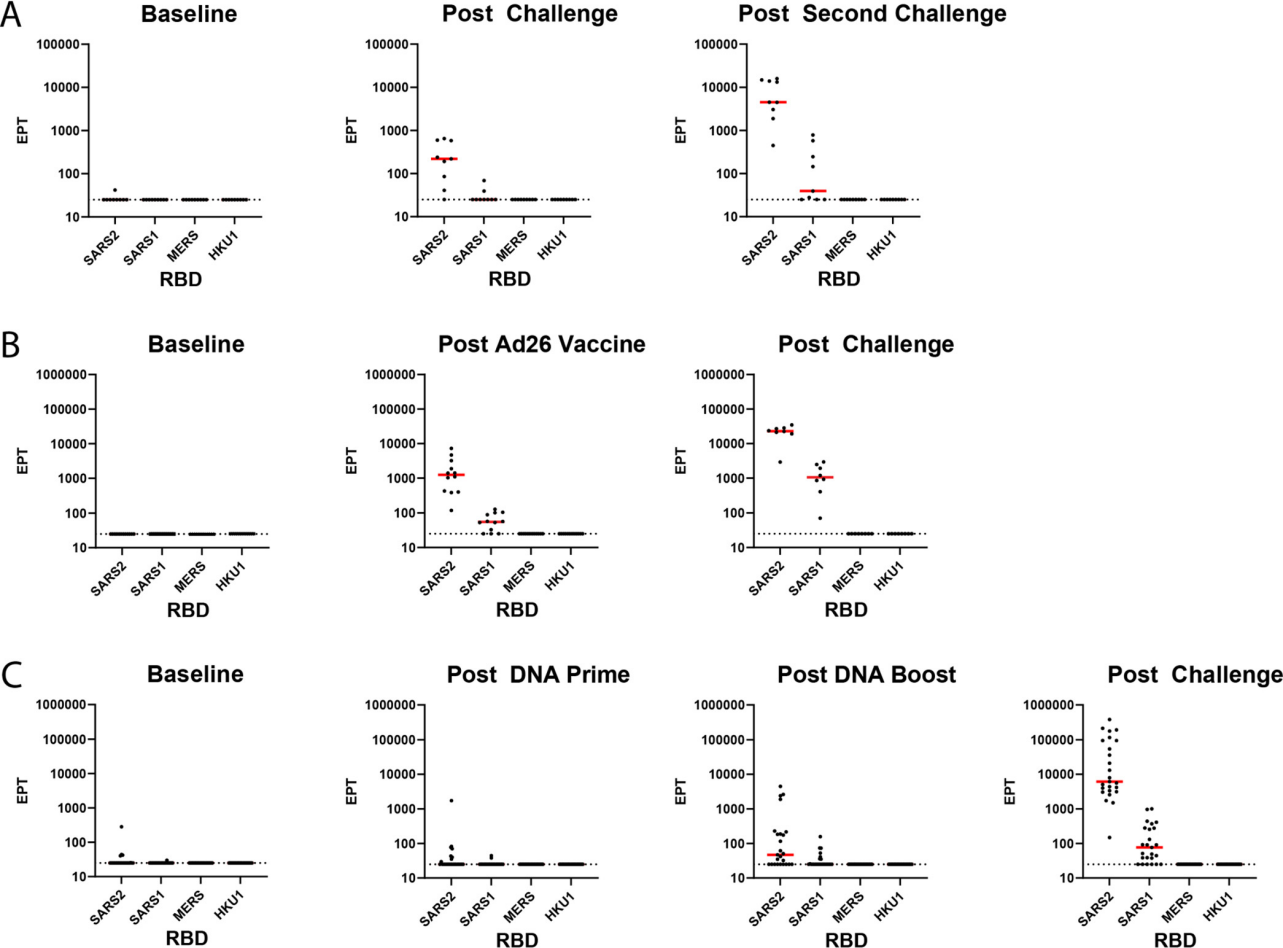
RBD ELISA to CoVs. Binding antibody ELISAs in NHPs challenged twice with SARS-CoV-2 virus (A), vaccinated with a SARS-CoV-2 spike-expressing Ad26 vaccine and then challenged with SARS-CoV-2 virus (B), or primed and boosted with SARS-COV-2 spike-expressing DNA vaccines and then challenged with SARS CoV-2 (C). Postvaccination and postchallenge time points are all ≥2 weeks after vaccination/challenge. The interpolated endpoint titers (EPT) are reported.

In the reinfection cohort, serum postinfection and reinfection showed increased reactivity to the SARS-CoV-2 RBD compared to baseline serum by ELISA (Fig. 3A). SARS-CoV-1 RBD reactivity was detectable in 2 of 9 NHPs after one infection and in 5 of 9 NHPs after two challenges (Fig. 3A). This same pattern occurred in the cohort of 12 NHPs vaccinated with Ad26 expressing SARS-CoV-2 S isoforms. All twelve Ad26-vaccinated NHPs had appreciable serum anti-SARS-CoV-2 RBD responses post-vaccination and generally showed increased responses to SARS-CoV-2 RBD post-challenge (Fig. 3B). Nine of twelve of these Ad26-vaccinated NHPs also had detectable SARS-CoV-1 RBD-binding titers post-vaccination, and SARS-CoV-1 RBD antibody-binding responses were detected in 8 of 8 NHPs post-challenge (Fig. 3B). Twenty-five NHPs were primed and boosted with DNA vaccines carrying various SARS-CoV-2 spike isoforms. Anti-SARS-CoV-2 RBD serum responses were detectable in eight of 25 vaccinated NHPs post DNA prime, 15 of 25 post DNA boost, and all 25 vaccinated NHPs postchallenge (Fig. 3C). Anti-SARS-CoV-1 RBD serum responses were detected in 3, 9, and 19 NHPs post prime, boost, and challenge, respectively (Fig. 3C). MERS-CoV and HKU1 RBD-binding antibodies were not detected in any of the infected, Ad26-vaccinated, or DNA-vaccinated NHPs in these cohorts at any time points (Fig. 3A to C).

### Full-length CoV S protein cross-reactivity.

**FIG 4:**
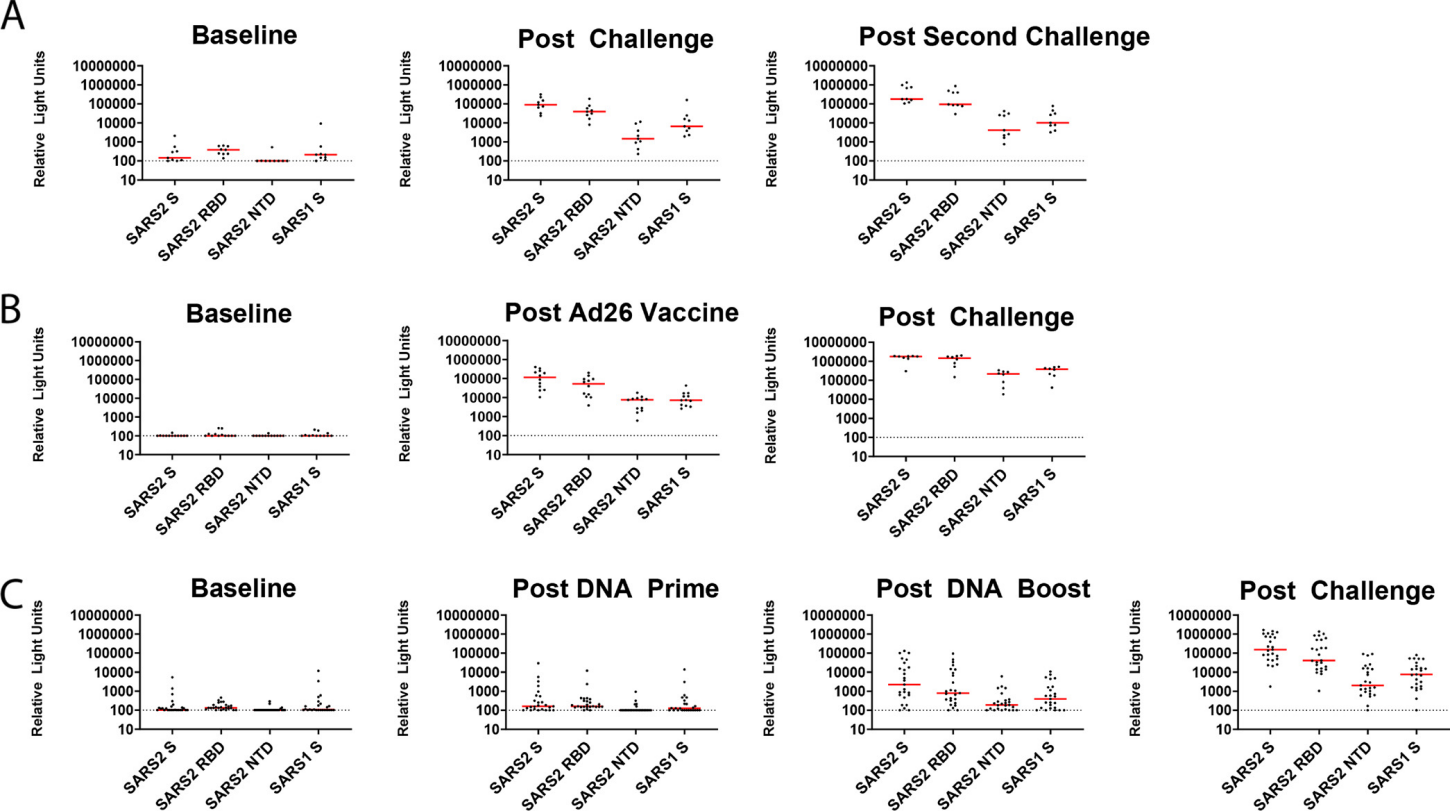
Mesoscale Discovery binding assays to SARS-CoV-1 and SARS-CoV-2. MSD antibody binding responses from NHPs challenged twice with SARS-CoV-2 virus (A), vaccinated with a SARS-CoV-2 spike-expressing Ad26 vaccine and then challenged with SARS-CoV-2 virus (B), or primed and boosted with SARS-CoV-2 spike-expressing DNA vaccines and then challenged with SARS CoV-2 (C). Responses were measured against the full-length spike (S) protein of SARS-CoV-2 (SARS2 S), the N-terminal domain of SARS-COV-2 spike (SARS-2 NTD), the RBD of SARS-CoV-2 Spike (SARS-2 RBD), and the S protein of SARS-CoV-1 (SARS1 S). Postvaccination and postchallenge time points are all ≥2 weeks after vaccination/challenge.

We next assessed the cross-reactivity of these serum responses to full-length CoV spike proteins of SARS-CoV-1, SARS-CoV-2, HKU1, OC43, NL64, 229E, as well as the N-terminal domain (NTD) and RBD of SARS-CoV-2 by electrochemiluminescence assays (ECLA) from MesoScale Discovery. This assay allowed for multiplexed detection of a panel of up to 9 antigens per serum sample in a single well. In the reinfection study, SARS-CoV-2 spike reactivity increased from baseline to post-infection and increased again post-rechallenge (Fig. 4A). Likewise, for the Ad26-vaccinated and challenged NHPs, the vaccinated NHPs showed increased SARS-CoV-2 spike reactivity post-vaccination compared to baseline and further increased SARS-CoV-2 spike responses post-challenge (Fig. 4B). Most of the DNA-vaccinated NHPs displayed modest increases in SARS-CoV-2 S responses post DNA boost, and all the DNA-vaccinated NHPs had robust SARS-CoV-2 spike reactivity post-challenge (Fig. 4C). SARS-CoV-1 spike reactivity was also detected in most animals with detectable SARS-CoV-2 spike reactivity, but at a lower level (Fig. 4). The responses to the RBD and NTD followed the same pattern of increased response with increased exposure in all the NHP cohorts (Fig. 4). These data show that S responses elicited by SARS-CoV-2 vaccines partially cross-react with SARS-CoV-1.

**FIG 5:**
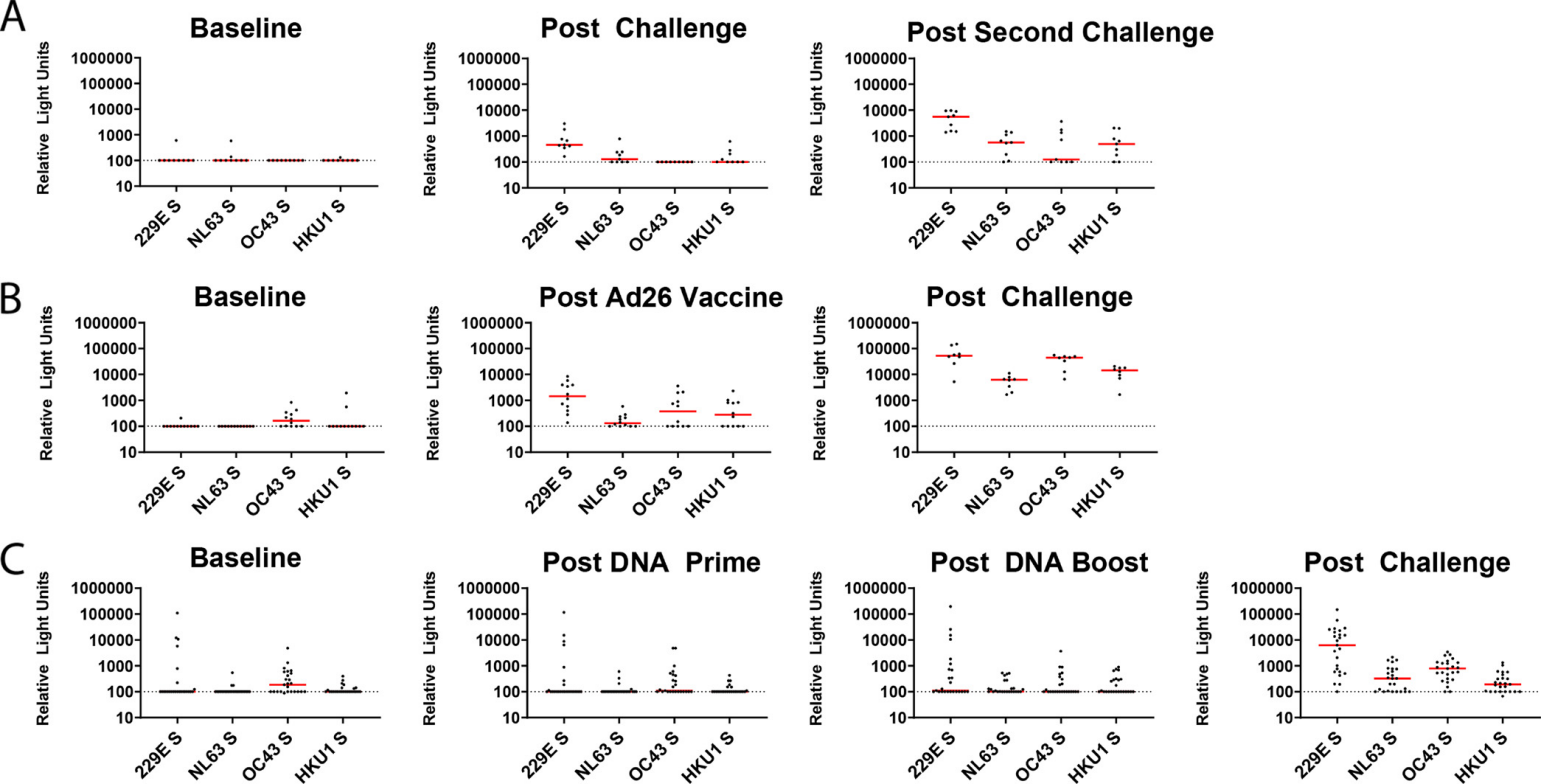
Mesoscale Discovery binding assays to common cold CoVs. MSD antibody binding responses from NHPs challenged twice with SARS-CoV-2 virus (A), vaccinated with a SARS-CoV-2 spike-expressing Ad26 vaccine and then challenged with SARS-CoV-2 virus (B), or primed and boosted with SARS-COV-2 spike-expressing DNA vaccines and then challenged with SARS-CoV-2 (C). Responses were measured against the full-length spike (S) protein of 229E, NL63, OC43, and HKU1. Postvaccination and postchallenge time points are all ≥2 weeks after vaccination/challenge.

The responses for the common cold hCoV spike proteins showed a similar pattern of reactivity. In the reinfection study, the NHPs showed increased reactivity to 229E, NL63, and HKU1 post-challenge compared to baseline, and these signals further increased post-reinfection, as did post-reinfection reactivity to OC43 (Fig. 5A). In the Ad26 vaccination study, we detected increased reactivity to all four hCoV S proteins post-vaccination and these signals further increased post-challenge (Fig. 5B). In the DNA vaccine cohort, we detected slightly increased reactivity post-boost to all hCoVs, and post-challenge reactivity to all four hCoV S proteins increased (Fig. 5C). The full-length spike proteins, as discussed, have higher structural and sequence homology than the specific RBDs due to the more highly conserved S2 region. This greater sequence conservation in the S2 domain likely accounts for the responses seen in infected, Ad26-vaccinated, DNA-vaccinated, and challenged NHPs to the hCoVs (Fig. 5).

### SARS-CoV-1 and SARS-CoV-2 pseudovirus neutralization.

**FIG 6:**
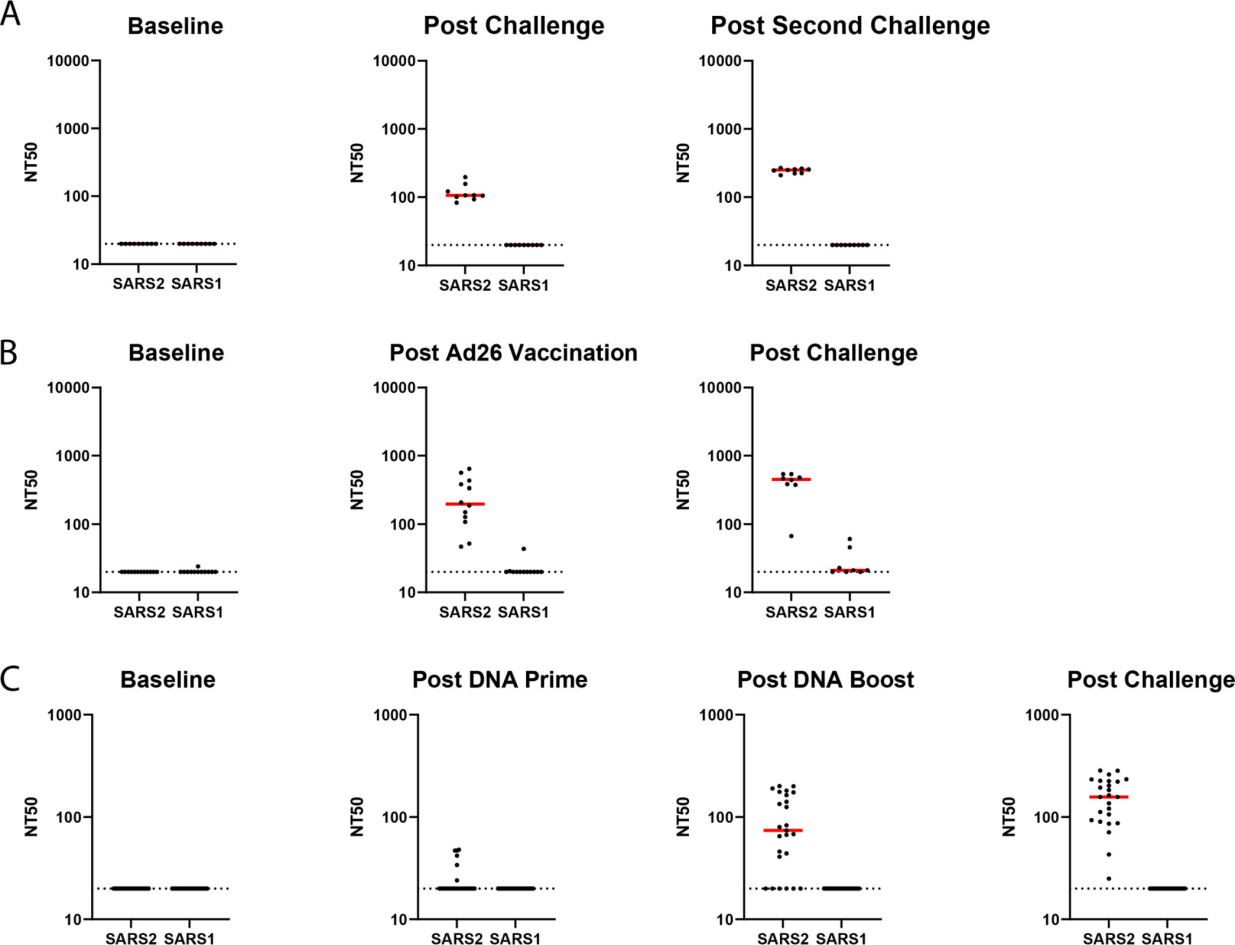
SARS-CoV-1 and SARS-CoV-2 pseudovirus neutralization assays. Pseudovirus neutralizing antibody titers (NT50) for SARS-CoV-1 and SARS-CoV-2 from NHPs challenged twice with SARS-CoV-2 virus (A), vaccinated with a SARS-CoV-2 spike-expressing Ad26 vaccine and then challenged with SARS-CoV-2 virus (B), or primed and boosted with SARS-CoV-2 spike-expressing DNA vaccines and then challenged with SARS-CoV-2 (C). Postvaccination and postchallenge time points are all ≥2 weeks after vaccination/challenge.

Current evidence suggests that RBD-directed antibodies are often potent neutralizing antibodies (21, 37, 38). We therefore assessed the pseudovirus neutralization potential of the SARS-CoV-2 and SARS-CoV-1 RBD-binding antibodies in SARS-CoV-2-infected and vaccinated macaques. We assessed both SARS-CoV-2 and SARS-CoV-1 pseudovirus neutralization using a lentivirus-based pseudovirus neutralization assay (Yu, J. et al., in press). Neutralizing responses to SARS-CoV-2 pseudovirus were detected in all of the infected and rechallenged NHPs (Fig. 6A) and all of the Ad26-vaccinated NHPs post-vaccination (Fig. 6B). Of the 25 DNA-vaccinated NHPs post DNA prime and DNA boost, 7 and 20, respectively, had detectable neutralizing responses, and all animals had neutralizing antibody responses post-challenge (Fig. 6C). SARS-CoV-1 pseudovirus neutralization was only detected in 1 of 12 NHPs post Ad26 vaccination (*P* = 0.0001 comparing SARS-CoV-2 and SARS-CoV-1 neutralization rates, two-sided Fisher’s exact test) and 2 of 8 NHPs after Ad26 vaccination and challenge (*P* = 0.007 comparing SARS-CoV-2 and SARS-CoV-1 neutralization rates, two-sided Fisher’s exact test) (Fig. 6B). The observed binding antibody titers directed against SARS-CoV-1 RBD or the full-length SARS-CoV-1 S protein therefore do not appear to translate to detectable SARS-CoV-1 pseudovirus neutralization.

## DISCUSSION

Here, we describe the cross-reactive CoV antibody profiles in NHPs following infection with SARS-CoV-2 and/or vaccination with SARS-CoV-2 S protein with DNA- or Ad26-based vaccines. These NHPs have previously been assessed for the binding, neutralizing, and non-neutralizing effector antibodies, as well as protective efficacy, following SARS-CoV-2 challenge (34–36). Ad26-based vaccination elicited the highest SARS-CoV-2 S protein- or RBD-directed antibody binding titers, followed by infection, followed by DNA-based vaccination, and all of these modalities then saw a boost in titers after challenge. We show that SARS-CoV-2 infection and vaccination resulted in similar patterns of cross-reactive antibody responses against SARS-CoV-1, but lower to other CoVs. Serum from these NHPs neutralized SARS-CoV-2 but only minimally SARS-CoV-1. Future studies may include expanded pseudovirus neutralization assays, but the lack of RBD-binding titers for coronaviruses outside the sarbecovirus family focused our efforts on SARS-CoV-2 and SARS-CoV-1 pseudovirus neutralization. Future studies will also assess immune responses in infected and vaccinated NHPs and humans to the recently emerged SARS-CoV-2 variants of concern.

In preclinical models, vaccines eliciting responses to SARS-CoV-2 S protein are protective against SARS-CoV-2 challenge; S binding and neutralizing antibodies have been reported to be correlates of protection (39). Our data suggest that SARS-CoV-2 S exposure, either in the context of natural infection or vaccination, results in cross-reactive antibody responses that recognize antigenically distinct CoVs, but at lower titers. The lack of neutralization of SARS-CoV-1 may be due to cross-reactive antibodies that recognize non-neutralizing sites on the SARS-CoV-1 RBD or lower-affinity antibodies. While these antibodies do not seem to be neutralizing, they also do not detectably enhance infectivity *in vitro* (39).

Understanding serum responses to multiple CoVs will be necessary for future pandemic preparedness, prediction of responses to CoVs circulating in animal reservoirs, and the potential development of pancoronavirus vaccines (18, 40, 41). Our NHP model allowed us to assess the influence of SARS-CoV-2 S exposure on other CoV-directed humoral responses. Further research will be necessary to address whether these responses are cross-reactive to other CoVs not included in our panel, or whether this pattern of cross-reactivity would hold true for initial exposure to a different CoV. Ongoing studies in COVID-19-convalescent humans and naïve donors show that SARS-CoV-2-uninfected children and young adults have circulating antibodies that target the SARS-CoV-2 S2 domain and can neutralize the virus (26). These antibodies likely arose from other hCoV infections, the rate of which is higher in this younger age group, and may be cross-protective (26). Additionally, SARS-CoV-2 infection can skew human antibody repertoires to recognize both SARS-CoV-2 and other shared CoV epitopes (27). Further studies focused on the CD4 and CD8 T cell responses in humans infected or uninfected with SARS-CoV-2 have detailed extensive cross-reactivity of CD4 responses in uninfected individuals to SARS-CoV-2 epitopes (42). Additional studies in our laboratory and other laboratories will assess cross-reactive T cell responses in infected and vaccinated NHPs and human donors, as well as binding and neutralizing antibody responses to the recently emerged SARS-CoV-2 variants of concern.

In conclusion, we demonstrate limited cross-reactive antibody responses to SARS-CoV-1 and hCoVs in rhesus macaques infected with SARS-CoV-2 or vaccinated with Ad26 or DNA vaccines expressing SARS-CoV-2 S protein. While these antibody responses largely did not neutralize SARS-CoV-1, they still highlight a substantial degree of cross-reactive binding antibodies among hCoVs. These findings extend our understanding of the serum immune responses to SARS-CoV-2 S protein and the cross-reactivity of humoral coronavirus recognition.

## MATERIALS AND METHODS

### Phylogenetic analysis.

Representative spike protein sequences for SARS-CoV-2, SARS-CoV-1, MERS-CoV, 229E, NL63, OC43, and HKU1 (GenBank accession numbers: YP_009724390.1, AAR86775.1, YP_009724390.1, ABB90529.1, APF29063.1, and AIX10763.1, ABD75513.1) were aligned using MUSCLE alignment in Geneious Prime software. This alignment was then used to generate a neighbor-joining consensus tree in Geneious Prime software. The percent amino acid sequence identity for the spike proteins and RBDs to SARS-CoV-2 spike and RBD were calculated using Geneious Prime pairwise alignment tools. The surface conservation was calculated using the ConSurf Database (29) to display amino acid conservation on the surface of the SARS-CoV-2 spike protein (6VYB) (30). The RBMs were highlighted as previously defined (31).

### Animals and study design.

Serum samples were derived from studies described previously (34–36). Briefly, for the reinfection study, nine outbred Indian-origin adult male and female rhesus macaques (Macaca mulatta), 6 to 12 years old, were randomly allocated to groups. All animals were housed at Bioqual, Inc. (Rockville, MD). The animals were inoculated with SARS-CoV-2 at a total dose of 1.1 × 10^6^ plaque forming units (PFU) (group 1; *n* = 3), 1.1 × 10^5^ PFU (group 2; *n* = 3), or 1.1 × 10^4^ PFU (group 3; *n* = 3). These doses were administered as 1 ml by the intranasal (i.n.) route (0.5 ml in each nare) and 1 ml by the intratracheal (i.t.) route. On day 35 following challenge, animals were rechallenged with SARS-CoV-2 at matched doses to those utilized in the initial challenge. For the Ad26 vaccination study, 22 outbred Indian-origin adult male and female rhesus macaques (Macaca mulatta), 6 to 12 years old, were randomly allocated to groups. All animals were housed at Bioqual, Inc. (Rockville, MD). Animals received Ad26 vectors expressing S.dTM.PP (*n* = 6), S.PP (*n* = 6), and sham controls (*n* = 10). Animals received a single immunization of 10^11^ viral particles (vp) Ad26 vectors by the intramuscular route without adjuvant at week 0. At week 6, all animals were challenged with 1.0 × 10^5^ 50 % tissue culture infective dose (TCID_50_) (1.2 × 10^8^ RNA copies, 1.1 × 10^4^ PFU) SARS-CoV-2. Virus was administered as 1 ml by the intranasal (i.n.) route (0.5 ml in each nare) and 1 ml by the intratracheal (i.t.) route. For the DNA vaccination study, 35 outbred Indian-origin adult male and female rhesus macaques (Macaca mulatta), 6 to 12 years old, were randomly allocated to groups. All animals were housed at Bioqual, Inc. (Rockville, MD). Animals received DNA vaccines expressing S protein (*n* = 4), S.dCT (*n* = 4), S.dTM (*n* = 4), S1 (*n* = 4), RBD (*n* = 4), S.dTM.PP (*n* = 5), and sham controls (*n* = 10). Animals received 5 mg of DNA vaccines at week 0 and week 3. At week 6, all animals were challenged with 1.0 × 10^5^ TCID_50_ (1.2 × 10^8^ RNA copies, 1.1 × 10^4^ PFU) SARS-CoV-2. Virus was administered as 1 ml by the intranasal (i.n.) route (0.5 ml in each nare) and 1 ml by the intratracheal (i.t.) route. All animal studies were conducted in compliance with all relevant local, state, and federal regulations and were approved by the Bioqual Institutional Animal Care and Use Committee (IACUC).

### ELISAs.

Enzyme-linked immunosorbent assay (ELISA) RBD-specific binding antibodies were assessed by ELISA essentially as described (34–36, 43). Briefly, 96-well plates were coated with 1μg/ml SARS-CoV-2 RBD, SARS-CoV-1 RBD, MERS-CoV RBD, or HKU1 RBD protein in 1× Dulbecco’s phosphate-buffered saline (DPBS) and incubated at 4°C overnight. After incubation, plates were washed once with 200 μl wash buffer (0.05% Tween 20 in 1× DPBS) and blocked with 350 μl casein block/well for 2 to 3 h at room temperature. After incubation, the blocking solution was discarded and plates were blotted dry. Serial dilutions of heat-inactivated serum diluted in casein block were added to wells and plates were incubated for 1 h at room temperature, prior to three further washes and a 1-h incubation with a 1:1,000 dilution of anti-macaque IgG horseradish peroxidase (HRP) (NIH NHP Reagent Program) at room temperature in the dark. Plates were then washed three times, and 100 μl of SeraCare KPL TMB SureBlue Start solution was added to each well; plate development was halted by the addition of 100 μl of SeraCare KPL TMB Stop solution per well. The absorbance at 450 nm was recorded using a VersaMax or Omega microplate reader. ELISA endpoint titers were defined as the highest reciprocal serum dilution that yielded an absorbance of >0.2. Log_10_ endpoint titers are reported.

### Pseudovirus neutralization assay.

The SARS-CoV-2 and SARS-CoV-1 pseudoviruses expressing a luciferase reporter gene were generated in an approach similar to that described previously (35, 36, 43). Briefly, the packaging construct psPAX2 (AIDS Resource and Reagent Program), luciferase reporter plasmid pLenti-CMV Puro-Luc (Addgene), and spike protein expressing pcDNA3.1-SARS CoV-2 SΔCT or pcDNA3.1-SARS CoV-1 for SARS-CoV-2 and SARS-CoV-1 pseudovirus generation, respectively, were cotransfected into HEK293T cells with calcium phosphate. The supernatants containing the pseudotype viruses were collected at 48 h post-transfection. Pseudotype viruses were purified by filtration with a 0.45 μm filter. To determine the neutralization activity of the antisera from vaccinated animals, HEK293T-hACE2 cells were seeded in 96-well tissue culture plates at a density of 1.75 × 10^4^ cells/well overnight. Two-fold serial dilutions of heat-inactivated serum samples were prepared and mixed with 50 μl of pseudovirus. The mixture was incubated at 37°C for 1 hour before adding to HEK293T-hACE2 cells. Forty-eight hours after infection, cells were lysed in the Steady-Glo luciferase assay (Promega) according to the manufacturer’s instructions. Neutralization titers were defined as the sample dilution at which a 50% reduction in relative light units (RLU) was observed relative to the average of the virus control wells.

### Electrochemiluninescence assays.

Mesoscale Discovery (MSD) coronavirus panel 2 plates for COVID-19 serology assays (K15369U-2) were designed and produced by Mesoscale Discovery with 9 antigen spots in each well. The antigens included were hCoV-229E spike, hCoV-NL63 spike, hCoV-OC43 spike, hCoV-HKU1 spike, SARS-CoV-1 spike, SARS-CoV-2 spike, SARS-CoV-2 spike RBD, SARS-CoV-2 NTD, and SARS-CoV-2 N protein. The plates were blocked with 50 μl of Blocker A solution for at least 30 min at room temperature with shaking at 700 rpm on a digital microplate shaker. During blocking, the serum was diluted 1:5,000 in Diluent 100. The plates were then washed 3 times with 150 μl of the MSD kit wash buffer, blotted dry, and 50 μl of the diluted samples was added in duplicate to the plates and set to shake at 700 rpm at room temperature for at least 2 h. The plates were again washed 3 times and 50 μl of SULFO-tagged anti-human IgG detection antibody diluted to 1× in Diluent 100 was added to each well and incubated with shaking at 700 rpm at room temperature for at least 1 h. Plates were then washed 3 times and 150 μl of MSD GOLD read buffer B was added to each well and the plates were read immediately after on a MESO QuickPlex SQ 120 machine. MSD titers for each sample were reported as relative light units (RLU), which were calculated as sample RLU minus blank RLU for each spot for each sample. The limit of detection was defined as 100 RLU for each assay.

## References

[1] Zhou P, Yang X-L, Wang X-G, Hu B, Zhang L, Zhang W, Si H-R, Zhu Y, Li B, Huang C-L. 2020. A pneumonia outbreak associated with a new coronavirus of probable bat origin. nature 579:270–273. 10.1038/s41586-020-2012-7. 32015507 PMC7095418

[2] Wu F, Zhao S, Yu B, Chen Y-M, Wang W, Song Z-G, Hu Y, Tao Z-W, Tian J-H, Pei Y-Y. 2020. A new coronavirus associated with human respiratory disease in China. Nature 579:265–269. 10.1038/s41586-020-2008-3. 32015508 PMC7094943

[3] Bio NSE, Pfizer. 2021. Study to describe the safety, tolerability, immunogenicity, and efficacy of RNA vaccine candidates against COVID-19 in healthy individuals. https://ClinicalTrials.gov/show/NCT04368728.

[4] ModernaTX, Inc., Biomedical Advanced Research and Development Authority, National Institute of Allergy and Infectious Diseases (NIAID). 2020. A study to evaluate efficacy, safety, and immunogenicity of mRNA-1273 vaccine in adults aged 18 years and older to prevent COVID-19. https://ClinicalTrials.gov/show/NCT04470427.

[5] Janssen Vaccines & Prevention B.V. 2020. A study of Ad26.COV2.S for the prevention of SARS-CoV-2-mediated COVID-19 in adult participants. https://ClinicalTrials.gov/show/NCT04505722.

[6] AstraZeneca, Iqvia Pty Ltd. 2020. Phase III double-blind, placebo-controlled study of AZD1222 for the prevention of COVID-19 in adults. https://ClinicalTrials.gov/show/NCT04516746.

[7] Butantan Institute, Sinovac Life Sciences Co., Ltd. 2021. Clinical trial of efficacy and safety of Sinovac's adsorbed COVID-19 (inactivated) vaccine in healthcare professionals. https://ClinicalTrials.gov/show/NCT04456595.

[8] CanSino Biologics Inc., Beijing Institute of Biotechnology. 2021. Phase III trial of a COVID-19 vaccine of adenovirus vector in adults 18 years old and above. https://ClinicalTrials.gov/show/NCT04526990.

[9] China National Biotec Group Company Limited, G42 Healthcare Company, Abu Dhabi Health Services Company, Wuhan Institute of Biological Products Co., Ltd., Beijing Institute of Biological Products Co., Ltd. 2021. A study to evaluate the efficacy, safety and immunogenicity of inactivated SARS-CoV-2 vaccines (Vero cell) in healthy population aged 18 years old and above. https://ClinicalTrials.gov/show/NCT04510207.

[10] PT Bio Farma, Faculty of Medicine Universitas Padjadjaran, National Institute of Health Research and Development, Ministry of Health Republic of Indonesia, Sinovac Life Sciences Co., Ltd. 2021. Efficacy, safety and immunogenicity study of SARS-CoV-2 inactivated vaccine (COVID-19). https://ClinicalTrials.gov/show/NCT04508075.

[11] Health Institutes of Turkey. 2021. Clinical trial for SARS-CoV-2 vaccine (COVID-19). https://ClinicalTrials.gov/show/NCT04582344.

[12] Laboratorio Elea Phoenix S.A., Beijing Institute of Biological Products Co., Ltd., China National Biotec Group Company Limited, Fundación Huésped. 2021. Clinical trial to evaluate the efficacy, immunogenicity and safety of the inactivated SARS-CoV-2 vaccine (COVID-19). https://ClinicalTrials.gov/show/NCT04560881.

[13] Novavax. 2021. A study looking at the effectiveness, immune response, and safety of a COVID-19 vaccine in adults in the United Kingdom. https://ClinicalTrials.gov/show/NCT04583995.

[14] Baden LR, El Sahly HM, Essink B, Kotloff K, Frey S, Novak R, Diemert D, Spector SA, Rouphael N, Creech CB. 2020. Efficacy and safety of the mRNA-1273 SARS-CoV-2 vaccine. N Engl J Med 384:403–416. 10.1056/NEJMoa2035389. 33378609 PMC7787219

[15] Polack FP, Thomas SJ, Kitchin N, Absalon J, Gurtman A, Lockhart S, Perez JL, Pérez Marc G, Moreira ED, Zerbini C. 2020. Safety and efficacy of the BNT162b2 mRNA covid-19 vaccine. N Engl J Med 383:2603–2615. 10.1056/NEJMoa2034577. 33301246 PMC7745181

[16] Voysey M, Clemens SAC, Madhi SA, Weckx LY, Folegatti PM, Aley PK, Angus B, Baillie VL, Barnabas SL, Bhorat QE. 2021. Safety and efficacy of the ChAdOx1 nCoV-19 vaccine (AZD1222) against SARS-CoV-2: an interim analysis of four randomised controlled trials in Brazil, South Africa, and the UK. The Lancet 397:99–111. 10.1016/S0140-6736(20)32661-1. PMC7723445 33306989

[17] Ye Z-W, Yuan S, Yuen K-S, Fung S-Y, Chan C-P, Jin D-Y. 2020. Zoonotic origins of human coronaviruses. Int J Biol Sci 16:1686–1697. 10.7150/ijbs.45472. 32226286 PMC7098031

[18] Andersen KG, Rambaut A, Lipkin WI, Holmes EC, Garry RF. 2020. The proximal origin of SARS-CoV-2. Nat Med 26:450–452. 10.1038/s41591-020-0820-9. 32284615 PMC7095063

[19] McIntosh K, Perlman S. 2010. Mandell, Douglas, and Bennett's principles and practice of infectious diseases. Elsevier, Philadelphia, PA.

[20] Zhou W, Wang W, Wang H, Lu R, Tan W. 2013. First infection by all four non-severe acute respiratory syndrome human coronaviruses takes place during childhood. BMC Infect Dis 13:433. 10.1186/1471-2334-13-433. 24040960 PMC3848659

[21] Barnes CO, West AP, Jr, Huey-Tubman KE, Hoffmann MA, Sharaf NG, Hoffman PR, Koranda N, Gristick HB, Gaebler C, Muecksch F. 2020. Structures of human antibodies bound to SARS-CoV-2 spike reveal common epitopes and recurrent features of antibodies. Cell 182:828–842.e16. 10.1016/j.cell.2020.06.025. 32645326 PMC7311918

[22] Klumpp-Thomas C, Kalish H, Drew M, Hunsberger S, Snead K, Fay MP, Mehalko J, Shunmugavel A, Wall V, Frank P. 2020. Standardization of enzyme-linked immunosorbent assays for serosurveys of the SARS-CoV-2 pandemic using clinical and at-home blood sampling. medRxiv 10.1101/2020.05.21.20109280. PMC7782755 33397956

[23] Estrada LD, Schultz-Cherry S. 2019. Development of a universal influenza vaccine. J Immunol 202:392–398. 10.4049/jimmunol.1801054. 30617121 PMC6327971

[24] Pancera M, Changela A, Kwong PD. 2017. How HIV-1 entry mechanism and broadly neutralizing antibodies guide structure-based vaccine design. Current Opin HIV AIDS 12:229. 10.1097/COH.0000000000000360. PMC5557343 28422787

[25] Turner JS, Zhou JQ, Han J, Schmitz AJ, Rizk AA, Alsoussi WB, Lei T, Amor M, McIntire KM, Meade P. 2020. Human germinal centres engage memory and naive B cells after influenza vaccination. Nature 586:127–132. 10.1038/s41586-020-2711-0. 32866963 PMC7566073

[26] Ng KW, Faulkner N, Cornish GH, Rosa A, Harvey R, Hussain S, Ulferts R, Earl C, Wrobel AG, Benton DJ. 2020. Preexisting and de novo humoral immunity to SARS-CoV-2 in humans. Science 370:1339–1343. 10.1126/science.abe1107. 33159009 PMC7857411

[27] Shrock E, Fujimura E, Kula T, Timms RT, Lee I-H, Leng Y, Robinson ML, Sie BM, Li MZ, Chen Y. 2020. Viral epitope profiling of COVID-19 patients reveals cross-reactivity and correlates of severity. Science 370:eabd4250. 10.1126/science.abd4250. 32994364 PMC7857405

[28] Walls AC, Park Y-J, Tortorici MA, Wall A, McGuire AT, Veesler D. 2020. Structure, function, and antigenicity of the SARS-CoV-2 spike glycoprotein. Cell 181:281–292. 10.1016/j.cell.2020.02.058. 32155444 PMC7102599

[29] Landau M, Mayrose I, Rosenberg Y, Glaser F, Martz E, Pupko T, Ben-Tal N. 2005. ConSurf 2005: the projection of evolutionary conservation scores of residues on protein structures. Nucleic Acids Res 33:W299–W302. 10.1093/nar/gki370. 15980475 PMC1160131

[30] Yi C, Sun X, Ye J, Ding L, Liu M, Yang Z, Lu X, Zhang Y, Ma L, Gu W. 2020. Key residues of the receptor binding motif in the spike protein of SARS-CoV-2 that interact with ACE2 and neutralizing antibodies. Cell Mol Immunol 17:621–630. 10.1038/s41423-020-0458-z. 32415260 PMC7227451

[31] Ou X, Guan H, Qin B, Mu Z, Wojdyla JA, Wang M, Dominguez SR, Qian Z, Cui S. 2017. Crystal structure of the receptor binding domain of the spike glycoprotein of human betacoronavirus HKU1. Nat Commun 8:15216. 10.1038/ncomms15216. 28534504 PMC5529671

[32] Okba NM, Müller MA, Li W, Wang C, GeurtsvanKessel CH, Corman VM, Lamers MM, Sikkema RS, de Bruin E, Chandler FD. 2020. Severe acute respiratory syndrome coronavirus 2− specific antibody responses in coronavirus disease patients. Emerg Infect Dis 26:1478–1488. 10.3201/eid2607.200841. 32267220 PMC7323511

[33] Chia WN, Tan CW, Foo R, Kang AEZ, Peng Y, Sivalingam V, Tiu C, Ong XM, Zhu F, Young BE. 2020. Serological differentiation between COVID-19 and SARS infections. Emerg Microbes Infect 9:1497–1505. 10.1080/22221751.2020.1780951. 32529906 PMC7473126

[34] Chandrashekar A, Liu J, Martinot AJ, McMahan K, Mercado NB, Peter L, Tostanoski LH, Yu J, Maliga Z, Nekorchuk M. 2020. SARS-CoV-2 infection protects against rechallenge in rhesus macaques. Science 369:812–817. 10.1126/science.abc4776. 32434946 PMC7243369

[35] Mercado NB, Zahn R, Wegmann F, Loos C, Chandrashekar A, Yu J, Liu J, Peter L, McMahan K, Tostanoski LH. 2020. Single-shot Ad26 vaccine protects against SARS-CoV-2 in rhesus macaques. Nature 586:583–588. 10.1038/s41586-020-2607-z. 32731257 PMC7581548

[36] Yu J, Tostanoski LH, Peter L, Mercado NB, McMahan K, Mahrokhian SH, Nkolola JP, Liu J, Li Z, Chandrashekar A. 2020. DNA vaccine protection against SARS-CoV-2 in rhesus macaques. Science 369:806–811. 10.1126/science.abc6284. 32434945 PMC7243363

[37] Hurlburt NK, Seydoux E, Wan Y-H, Edara VV, Stuart AB, Feng J, Suthar MS, McGuire AT, Stamatatos L, Pancera M. 2020. Structural basis for potent neutralization of SARS-CoV-2 and role of antibody affinity maturation. Nature Commun 11:5413. 10.1038/s41467-020-19231-9. 33110068 PMC7591918

[38] Seydoux E, Homad LJ, MacCamy AJ, Parks KR, Hurlburt NK, Jennewein MF, Akins NR, Stuart AB, Wan Y-H, Feng J. 2020. Analysis of a SARS-CoV-2-infected individual reveals development of potent neutralizing antibodies with limited somatic mutation. Immunity 53:98–105. 10.1016/j.immuni.2020.06.001. 32561270 PMC7276322

[39] McMahan K, Yu J, Mercado NB, Loos C, Tostanoski LH, Chandrashekar A, Liu J, Peter L, Atyeo C, Zhu A, Bondzie EA, Dagotto G, Gebre MS, Jacob-Dolan C, Li Z, Nampanya F, Patel S, Pessaint L, Van Ry A, Blade K, Yalley-Ogunro J, Cabus M, Brown R, Cook A, Teow E, Andersen H, Lewis MG, Lauffenburger DA, Alter G, Barouch DH. 2020. Correlates of protection against SARS-CoV-2 in rhesus macaques. Nature 590:630–634. 10.1038/s41586-020-03041-6. 33276369 PMC7906955

[40] Boni MF, Lemey P, Jiang X, Lam TT-Y, Perry B, Castoe T, Rambaut A, Robertson DL. 2020. Evolutionary origins of the SARS-CoV-2 sarbecovirus lineage responsible for the COVID-19 pandemic. Nat Microbiol 5:1408–1417. 10.1038/s41564-020-0771-4. 32724171

[41] Menachery VD, Yount BL, Sims AC, Debbink K, Agnihothram SS, Gralinski LE, Graham RL, Scobey T, Plante JA, Royal SR. 2016. SARS-like WIV1-CoV poised for human emergence. Proc Natll Acad Sci U S A 113:3048–3053. 10.1073/pnas.1517719113. PMC4801244 26976607

[42] Grifoni A, Weiskopf D, Ramirez SI, Mateus J, Dan JM, Moderbacher CR, Rawlings SA, Sutherland A, Premkumar L, Jadi RS. 2020. Targets of T cell responses to SARS-CoV-2 coronavirus in humans with COVID-19 disease and unexposed individuals. Cell 181:1489–1501. 10.1016/j.cell.2020.05.015. 32473127 PMC7237901

[43] Tostanoski LH, Wegmann F, Martinot AJ, Loos C, McMahan K, Mercado NB, Yu J, Chan CN, Bondoc S, Starke CE. 2020. Ad26 vaccine protects against SARS-CoV-2 severe clinical disease in hamsters. Nat Med 26:1694–1700. 10.1038/s41591-020-1070-6. 32884153 PMC7671939

